# Electrotunable liquid sulfur microdroplets

**DOI:** 10.1038/s41467-020-14438-2

**Published:** 2020-01-30

**Authors:** Guangmin Zhou, Ankun Yang, Yifei Wang, Guoping Gao, Allen Pei, Xiaoyun Yu, Yangying Zhu, Linqi Zong, Bofei Liu, Jinwei Xu, Nian Liu, Jinsong Zhang, Yanxi Li, Lin-Wang Wang, Harold Y. Hwang, Mark L. Brongersma, Steven Chu, Yi Cui

**Affiliations:** 1 Department of Materials Science and Engineering, Stanford University, Stanford, CA 94305 USA; 2 Shenzhen Geim Graphene Center, Tsinghua-Berkeley Shenzhen Institute & Tsinghua Shenzhen International Graduate School, Tsinghua University, Shenzhen, 518055 China; 3 Materials Sciences Division, Lawrence Berkeley National Laboratory, Berkeley, CA 94720 USA; 4 School of Chemical & Biomolecular Engineering, Georgia Institute of Technology, Atlanta, GA 30332 USA; 5 Stanford Institute for Materials and Energy Sciences, SLAC National Accelerator Laboratory, 2575 Sand Hill Road, Menlo Park, CA 94025 USA; 6 Department of Physics, Stanford University, Stanford, CA 94305 USA; 7 Department of Molecular and Cellular Physiology, Stanford University, Stanford, CA 94303 USA

**Keywords:** Electrochemistry, Materials chemistry, Optical materials and structures

## Abstract

Manipulating liquids with tunable shape and optical functionalities in real time is important for electroactive flow devices and optoelectronic devices, but remains a great challenge. Here, we demonstrate electrotunable liquid sulfur microdroplets in an electrochemical cell. We observe electrowetting and merging of sulfur droplets under different potentiostatic conditions, and successfully control these processes via selective design of sulfiphilic/sulfiphobic substrates. Moreover, we employ the electrowetting phenomena to create a microlens based on the liquid sulfur microdroplets and tune its characteristics in real time through changing the shape of the liquid microdroplets in a fast, repeatable, and controlled manner. These studies demonstrate a powerful in situ optical battery platform for unraveling the complex reaction mechanism of sulfur chemistries and for exploring the rich material properties of the liquid sulfur, which shed light on the applications of liquid sulfur droplets in devices such as microlenses, and potentially other electrotunable and optoelectronic devices.

## Abstract (web summary)

Manipulating liquids with tunable shape and optical functionalities in real time remains a great challenge. Here, the authors demonstrate electrotunable liquid sulfur microdroplets in an electrochemical cell and tune its characteristics in a fast, repeatable, and controlled manner.

## Introduction

There is a great demand to manipulate liquids with tunable shape, size, and motion for applications in electronic displays, optical switching, and electroactive flow devices^1–3^. Significant advances have been achieved through engineering micro/nanostructured surfaces to enhance liquid-repelling or wetting capabilities, such as designing ordered arrays of nanopits/nanopillars^4^, asymmetric nanostructured surfaces^5^, and tapered conical nanotextures^6^ to adjust the hydrophilic/hydrophobic properties. Other approaches to dynamically tune or actuate liquids such as pH-responsive microstructured surfaces^7^, thermal stimulated homeostasis system^8^, and magnetically-tunable structures^9^ have been designed to allow for spatial control of liquid movement under acid/base, temperature oscillation, and magnetic field conditions. However, the necessity for complex surface structure design/modification or irreversible transformation and slow response time are still challenges that need to be addressed.

Electrowetting is another facile and effective strategy to manipulate liquid by controlling the liquid wettability on a surface via applying an external electric field with the advantages of fast response and simple implementation without the need of complex surface structuring and patterning (Supplementary Fig. 1a). Therefore, it has been applied in a variety of fields such as micro/nano-fluidics, adaptive microlenses, etc^10,11^. Normally, a minimum of several tens of volts is required to apply across a dielectric layer to drive these devices^12^. A reduction in the operating voltage could greatly decrease the energy consumption of the whole system due to its voltage-squared dependence. Reducing the thickness of the dielectric layer leads to a lower required voltage; e.g., reversible electrowetting was achieved at < 50 V on an amorphous fluoropolymer dielectric layer thinner than 1 μm^13^, while an applied voltage of 15 V was sufficient to electrowet a thin (70 nm) dielectric layer with a fluoropolymer coating^14^. Recently, ultra-low-voltage electrowetting systems based on the interface between two immiscible electrolytic solutions^15^ or using graphite surfaces^16,17^ have been reported, reducing the voltage requirement to < 2.0 V, showing their great promise. However, despite these efforts, realization of a low activating voltage requires an extremely thin dielectric layer, which is challenging. In addition, previous studies have mainly investigated the wettability of water/oil on various insulating substrates (dielectric layers)^11^. To the best of our knowledge, active control of liquid wettability on a conducting surface has rarely been investigated so far.

Recently, we have discovered the phenomenon of supercooled liquid sulfur droplets well below its melting point (m.p. = 115.2 °C, supercooled liquid sulfur @ −40 °C) in electrochemical lithium sulfur (Li–S) battery cells^18^. Understanding of the kinetics and dynamics of the whole sulfur evolution is still in its infancy. In addition, in Li–S batteries, sulfur cathode performance is strongly related to the morphology, size, and distribution of sulfur during the nucleation, growth, and dissolution processes^19,20^. The understanding of these sulfur behaviors can provide insights on the design of advanced electrodes for high performance Li–S batteries.

Here, we propose a new mechanism to tune the wetting of a liquid droplet directly on a conducting substrate without a dielectric layer, and the system is operated with extremely low voltages (Supplementary Fig. 1b). We capture in situ observations of the dynamics of the liquid sulfur droplets and their low-voltage (< 4.0 V) electrowetting. The comprehensive investigation of the nucleation and growth behavior of sulfur droplets provides a full understanding of their nuclei size, density, and growth dynamics, in good agreement with classical nucleation and growth theory^21,22^. Furthermore, combining computational and experimental efforts, we achieved selective growth of sulfur droplets with controllable shape and distribution. Finally, we established an approach to create adjustable liquid microlenses and present electrotunable optical imaging. Our findings enable active control of the wettability of sulfur on conducting surfaces, open a variety of possibilities beyond common wettability control architectures such as wetting of water/oil on insulating substrates, and broaden the applications of supercooled liquid materials.

## Results

### In situ observation of sulfur nucleation

**Fig. 1:**
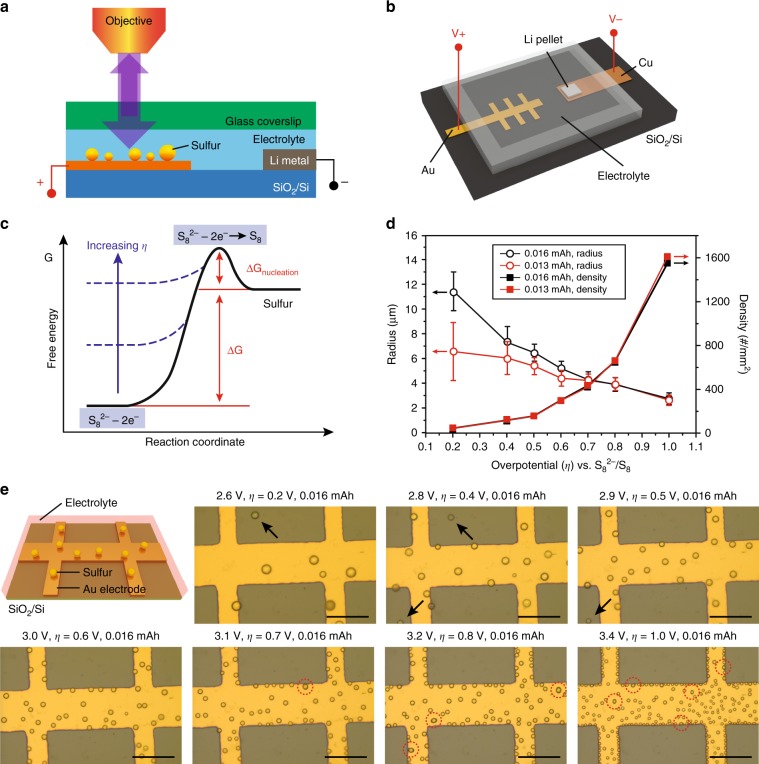
In situ observation of sulfur nucleation. **a** Schematic of the setup for the in situ optical observation. **b** Schematic of the assembled electrochemical device. **c** Free energy schematic showing the effects of increasing overpotential (*η*) on the energy barrier for sulfur nucleation. **d** Plot of sulfur droplet radius and density versus overpotential (*η*) at capacities of 0.013 and 0.016 mAh. Radius is measured for sulfur droplets after charging 0.013 and 0.016 mAh. **e** Optical images of sulfur droplets produced at different overpotentials with a fixed total charge capacity of 0.016 mAh. Scale bar in (**e**) is 100 μm.

Figure 1a shows the optical battery setup we designed for in situ optical observations of the sulfur evolution processes. Gold electrodes are fabricated on SiO_2_/Si substrates by depositing Ti/Au (3/50 nm) through a deposition mask. Li metal was laminated onto a copper foil and used as the counter electrode. The Au electrodes, Li metal, and the liquid electrolyte (Li_2_S_8_ dissolved in dioxolane/dimethyl ether (DOL/DME, 1:1 v/v) with 1 M LiTFSI salt and 1 wt% LiNO_3_ additive) were assembled in an Ar-filled glovebox and sealed by a thermoplastic film (Fig. 1b, see Experimental Section for details). LiNO_3_ was added to passivate the Li metal surface and suppress its reaction with polysulfide^23^. The transparent glass coverslip placed over the electrodes enabled in situ observations. The assembled device exhibits an open-circuit voltage (OCV) of 2.3–2.4 V with respect to Li/Li^+^. According to classical nucleation theory, the transformation of polysulfides to heterogeneously-nucleated elemental sulfur needs to overcome the free energy barrier related to the kinetics of the critical sulfur clusters^21,22,24–26^, which can be overcome by increasing the overpotential of the polysulfide oxidation processes (Fig. 1c). As discussed in an earlier work^18^, the electrochemical potential is the electrochemical equivalent to temperature as an extensive variable that determines in the nucleation and growth of condensation droplets. Figure 1d shows the radius of sulfur droplets as a function of the overpotential *η* with respect to S_8_^2−^/S_8_ (an equilibrium potential of about 2.4 V was established as shown in Supplementary Fig. 2; the in situ videos of nucleation under voltages 2.8 V and 3.4 V were selected to show in Supplementary Movie 1, 2). The radius of the sulfur droplets decreased with increasing overpotentials for both total charge capacities of 0.013 and 0.016 mAh (nominal capacity for the whole cell). For example, the radius decreased from around 11 μm at an overpotential of 0.2 V to 3 μm when the overpotential reached 1.0 V for a charge capacity of 0.016 mAh. Meanwhile, the number density increased significantly when the overpotential changed from 0.2 to 1.0 V. We found that the radius was inversely proportional to the overpotential and the areal number density of nuclei was proportional to the cubic power of overpotential (*η*^*3*^), consistent with classical nucleation and growth theory^21,22,24–26^ (Supplementary Fig. 3). Figure 1e shows the optical images of sulfur droplets acquired at different overpotentials ranging from 0.2 to 1.0 V with a fixed total charge capacity of 0.016 mAh. A clear dependence of size and nuclei density on the overpotential was observed. Spherical liquid sulfur droplets with larger size and lower density were produced on the electrode surface at small overpotentials while sulfur droplets with smaller size and higher density were produced at large overpotentials. Besides the sulfur droplets formed on the conducting Au electrodes, importantly we also observed a few droplets on the surface of insulating SiO_2_/Si (indicated by the arrows, Fig. 1e), which suggests that the sulfur can be electrochemically generated, dissolved in solution and precipitated out onto non-conducting substrate. Consistent with our previous results^18^, this observation is distinct from the traditional surface redox process where polysulfide anions transfer electrons to the metal electrodes and sulfur precipitates at the conducting surface.

### Sulfur wetting, growth, and de-wetting processes

**Fig. 2:**
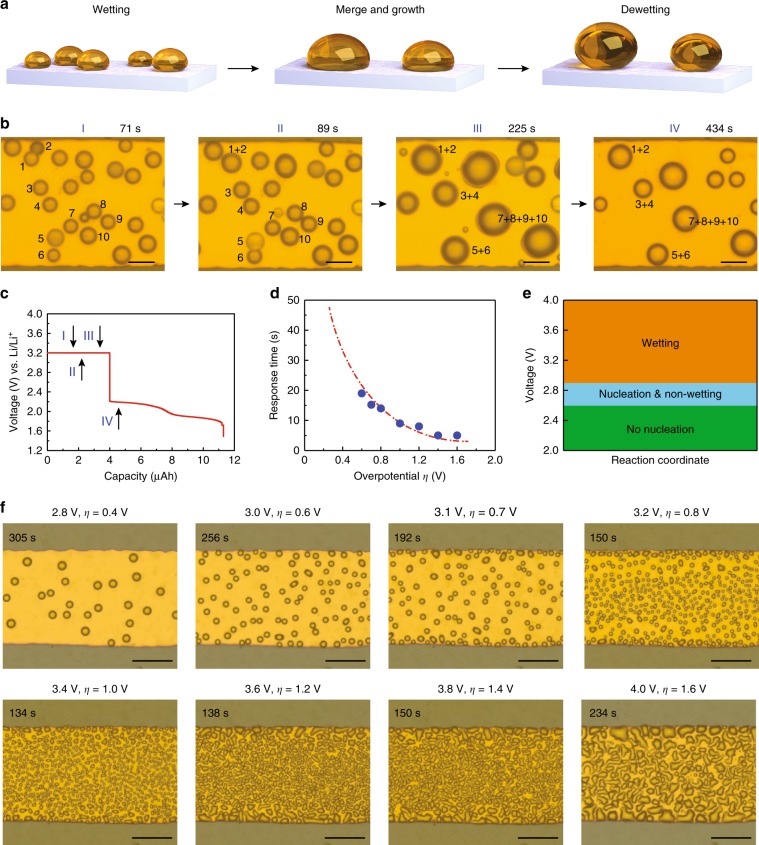
Direct observation of sulfur wetting, growth, and de-wetting processes. **a** Schematic illustration of the wetting, merging, growth and de-wetting processes of the sulfur droplets on gold electrode. **b** In situ real-time imaging and **c** corresponding voltage versus capacity curves for the wetting, merging, growth and de-wetting processes. The numbers shown in (**b**) refer to the droplets which will merge and grow. Scale bar in (**b**) is 20 μm. **d** Plot of the sulfur droplet response time versus overpotential (*η*). The response time refers to the shape change of the droplets from the wetting state to a near-spherical shape. The dashed line is a guide for the eyes. **e** Voltage-dependent no-nucleation range (below 2.6 V), nucleation but non-wetting range (2.6–2.9 V) and wetting range (higher than 2.9 V). **f** Snapshots of sulfur droplet wetting on gold at different voltages for a fixed total capacity of 0.001 mAh. Scale bar in (**f**) is 50 μm.

Interestingly, we also observed that some spherical droplets deformed into elliptic shapes (Fig. 1e, 3.1–3.4 V, circled by the red dashed lines), showing the wetting and merging behaviors of the sulfur droplets, especially at high voltages (i.e., large overpotentials). In order to elucidate the effect of overpotential on the wetting, growth, and de-wetting of sulfur droplets (Fig. 2a), the detailed evolution process was investigated by capturing optical images at different time and stages (Fig. 2b, see Supplementary Movie 3). The corresponding characteristic voltage profile is shown in Fig. 2c. When applying a constant voltage at 3.2 V versus Li/Li^+^, sulfur droplets started to wet the surface of the gold electrode, and the shape of sulfur changed from spheres (Fig. 2b I) to ellipsoids (Fig. 2b II, III). The numbers in Fig. 2b illustrate the merging of liquid sulfur droplets into larger droplets as more sulfur was deposited onto the gold electrode. When the voltage was released, the sulfur droplets rapidly changed back to their original round shape (Fig. 2b IV), indicative of induced dipole forces acting on the droplets during the reversible wetting/de-wetting processes. The droplets showed an obvious decrease in diameter, confirming the de-wetting phenomena (Supplementary Fig. 4). The electrowetting voltage is thus around 3.0 V, much lower than previous results for water-based electrowetting systems^13,14^. Figure 2d shows that the response time (from the wetted hemispherical state to a spherical shape) of the sulfur droplets is strongly related to the overpotential (driving force). The response time quickly reduced to around 5 s at a high constant voltage of 4.0 V (*η* *=* 1.6 V). The voltage window can be divided into the following three regions (Fig. 2e). At low voltage (2.0–2.6 V) with small overpotential, no sulfur nuclei are seen on the electrode surface, indicating insufficient driving force to oxidize polysulfides and overcome the nuclei energy barrier. As we increased the voltage to the range of 2.6–2.9 V, sulfur was produced but with no obvious wetting phenomenon. For voltages above 2.9 V, the sulfur droplets exhibited wetting and merging behaviors. The snapshots of real-time sulfur droplet wetting at different voltages for a fixed total capacity of 0.001 mAh are shown in Fig. 2f. The sulfur droplets formed sphere-like morphologies at low voltages with small overpotential (2.8 V, *η* *=* 0.4 V), but became noncircular in shape due to the electrowetting phenomenon when the overpotential was increased, and finally fused together to form island-like morphologies (e.g., 4.0 V, *η* *=* 1.6 V) due to the coalescence of multiple sulfur droplets. Supplementary Fig. 5 shows the complete time-series of snapshots for real-time sulfur droplet evolution, including wetting and de-wetting, at 4.0 V for a charge capacity of 0.0015 mAh as well as the dissolution processes when discharging to 1.0 V at a current of 0.05 mA (Supplementary Movie 4). In order to observe the side view of the droplets, we assembled a gold-coated nickel transmission electron microscopy (TEM) grid into our optical cell and generated liquid sulfur droplets on both the sidewalls and the top surface of the grid. Supplementary Fig. 6 shows the side view of the droplets when applying and releasing voltage. Similar to the surface, the sulfur droplets on the sidewalls changed wetting angle after applying voltage and changed back after releasing voltage, especially at voltages between 3.2 and 4.0 V. When holding at 4.0 V, the droplets exhibit small wetting angle, and after the voltage was released, the sulfur droplets rapidly changed back to their original round shape (Supplementary Fig. 6f), showing the electric field driven wetting phenomena.

### Selective wetting and mechanism understanding

**Fig. 3:**
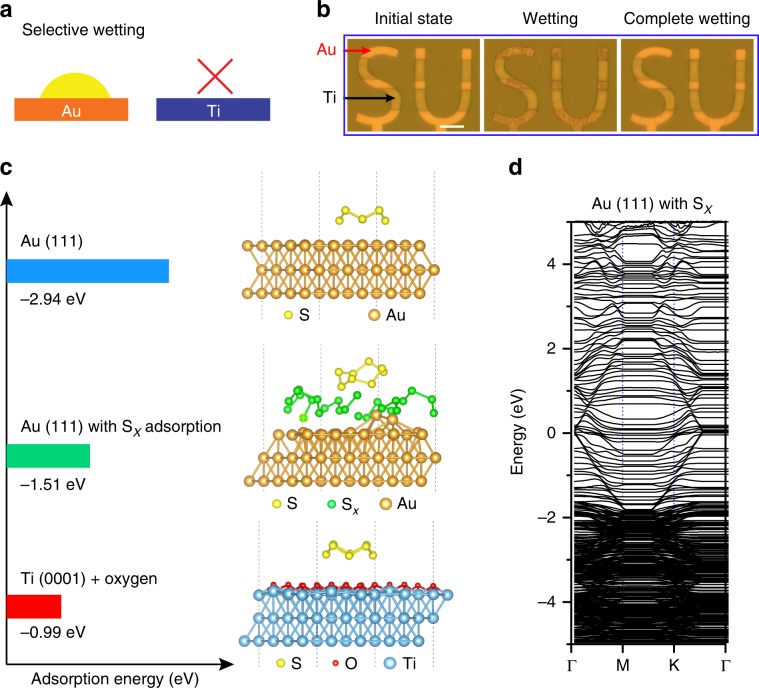
Selective wetting and mechanism understanding. **a** Schematic and **b** patterned “SU” design illustrating substrate-dependent electrowetting of sulfur on gold and titanium. Scale bar in (**b**) is 10 μm. **c** Adsorption energy and configuration of S_8_ adsorbed on the surface of gold (111), gold (111) covered with S_x_ layer and titanium (0001) covered by one layer of oxygen. **d** Band gap of gold (111) covered with S_x_ layer.

In contrast to the gold surfaces which can be wet by liquid sulfur (Supplementary Fig. 7), we did not observe any sulfur growth on the surface of titanium (Fig. 3a and Supplementary Fig. 8) over the entire range of applied voltage (from 2.4 to 4.0 V). Generally, a surface with a combination of both hydrophilic and hydrophobic properties at different regions is desirable for many applications, such as displays, electronic paper, microfluidic, and micro-optics devices^27^. Analogously, a surface with sulfiphilic (strong affinity for sulfur) and/or sulfiphobic (weak/no affinity for sulfur) properties can be designed and find potential applications in Li–S batteries. Here, an “SU” pattern with alternate gold and titanium coverage has been designed to show the successful selective wetting of liquid sulfur on the substrate (Fig. 3b, Supplementary Movie 5). Liquid sulfur droplets started to appear from the areas coated with gold (yellow color) once the voltage (4.0 V) was applied and quickly expanded to the entire gold patterned areas. However, no change was observed on the striped areas coated with titanium (dark green color), confirming the substrate-dependent electrowetting properties of sulfur. These results provide insights on the role of the current collectors in determining the sulfur generation and offer the platform to evaluate the correlation between the sulfur state and battery performance, which will advance electrode designs for high performance Li–S batteries.

To gain insight into the mechanism of the selective nucleation and wetting processes at atomic level, S_8_ adsorption properties on the surface of titanium (0001) and gold (111) were compared using density functional theory (DFT) calculations (Fig. 3c). Since titanium will be covered by a 1–2 nm thick passive oxide coating under ambient conditions^28^, titanium (0001) covered by one layer of oxygen was constructed to simulate the adsorption behavior of a S_8_ ring. Anatase TiO_2_ was used to obtain the conductivity properties of the passive oxide coating. The weak adsorption energy (−0.99 eV) that mainly attributes to the van der Waals interactions between S_8_ and the Ti–O layer indicates low affinity of S_8_ to the Ti–O layer. Furthermore, the passive oxide coating with a large band gap of 3.21 eV (Supplementary Fig. 9a) blocks the electron transfer to sulfur species and hampers the following redox chemistry. Therefore, sulfur nuclei cannot be produced on the surface of titanium. To verify our theoretical explanation, a higher voltage over 4.0 V (e.g., 4.5 V) was applied, where electron tunneling can occur and sulfur droplets were observed on the surface of titanium (Supplementary Fig. 10). In contrast, the adsorption energy of S_8_ on Au (111) is as high as −2.94 eV (Fig. 3c), indicating that sulfur will not only nucleate but also immediately wet the gold surface. However, based on the calculations, the S_8_ ring is unstable on the clean Au (111) surface and will decompose into two more stable S_4_ chains on Au (111) (Supplementary Fig. 11). On the other hand, atomically-distributed sulfur atoms on Au (111) will form stable sulfur chains (S_x_, 1 < *x* < 8) after relaxing (Supplementary Fig. 11), indicating that the S_x_ will not further decompose into atomic sulfur. Therefore, a layer of S_x_ possibly forms on Au (111) before the adsorption of S_8_ rings, which weakens the interaction between S_8_ and the gold surface (−1.51 eV, Fig. 3c). We also consider the conductivity of Au (111) and its surface covered with a S_x_ layer, and DFT calculations reveal that a band gap of gold was not opened by the S_x_ coating layer (Fig. 3d and Supplementary Fig. 9b). Based on these discussions, the chemical adsorption of S_8_ on the electrode surface and good electric conductivity are two key factors for the realization of successful wetting and hence selective wetting can be easily designed and achieved.

### Electrotunable liquid microlenses

**Fig. 4:**
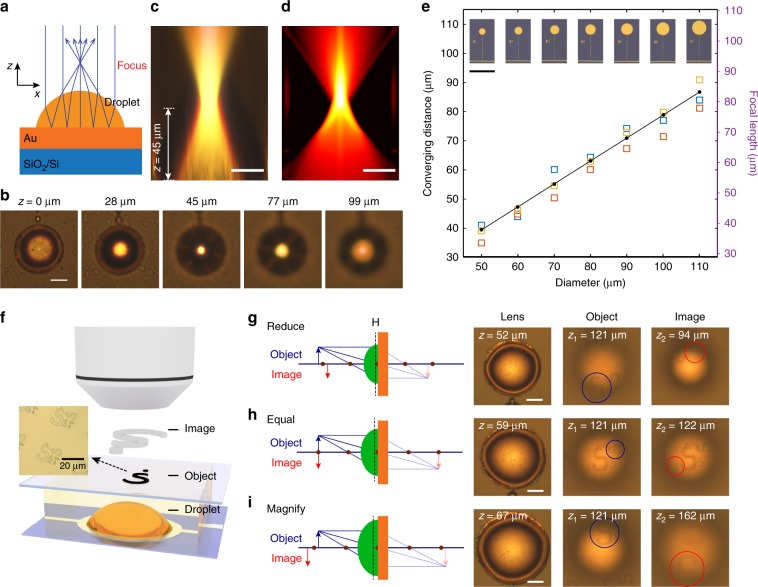
Electrotunable liquid microlenses. **a** Schematic and **b** optical microscopy images of a microlens based on a 60-µm sulfur droplet with a converging distance of 45 µm. **c** Measured intensity profile with cross sections in the xy plane. **d** Calculated intensity profile from ray tracing, scale bar in (**b**–**d**) is 20 µm. **e** The focal length of the microlenses can be tuned as a function of the diameter of the sulfur droplets, scale bar in (**e**) is 200 µm. The squares are experimental data and the black dots are simulated data. **f** Imaging of the letter “S” patterned on a cover glass slide with sulfur droplet-based microlenses, with **g** reduced, **h** equal, and **i** magnified images. The dashed lines in the schematics denoted by H indicate the location of the principal plane of the microlenses. scale bar in (**g**–**i**) is 20 µm.

The selective nucleation and wetting of liquid sulfur droplets from the original polysulfide electrolyte opens new opportunities for applications in electro-optics. Previously, tunable droplet-based microlenses were implemented by changing the curvature of the meniscus between two immiscible liquids, through electrowetting or adding surfactants^2,29^. Here we propose a new approach to form liquid microlenses with a single liquid, based on the electrochemical growth of sulfur. With moderate voltages (< 4.0 V) applied to the gold disk, well-defined sulfur microdroplets are formed^15^. The combination of induced dipole forces and electrostatic interactions of S_x_ molecules with a gold substrate allow the wetting angle, and hence the optical lens properties, to be tuned. The droplet-electrolyte interface refracts the incident light twice before it is focused to a single spot, with light paths shown in Fig. 4a. The incident, quasi-collimated light wave has incident angles smaller than 2° with the substrate normal. The voltage to create the droplet is considerably smaller than those used for common electrotunable liquid lenses^30^. The focusing ability of a microlens is measured by taking a series of optical images at different height and matches well with ray tracing calculations (Fig. 4b–d). Since the measurement is performed with an air objective, all the displacement in the *z* direction needs to be multiplied by *n*_*e*_, which is the measured refractive index of the electrolyte (Supplementary Note 1, Supplementary Fig. 12). The sulfur droplet $\left( {n_s = 2.0} \right)$ shown in the figure has a diameter of 60 µm, which creates a focal point with a 45 µm converging distance. Here the converging distance is defined as the distance between the focal point and substrate, different from focal length, which is defined as the distance between the focal point and the principal plane. In addition, the shape of the liquid sulfur droplets can be reconfigured by switching the external electrical field on or off, resulting in two different focal points (Supplementary Fig. 13). There are only small hysteretic behaviors when the microlens was re-created, for example, the focal length changed from 41 µm (voltage on) to 22 µm (voltage off) and then to 39 µm (voltage on again) (Supplementary Fig. 13b). The shape of the droplet with applied voltage is measured to be a spherical cap smaller than a half sphere for all sizes of gold disks we patterned (Supplementary Note 2, Supplementary Fig. 14), due to the selective sulfur growth. Therefore, by simply patterning gold disks with different sizes, we are able to control the curvature of the sulfur droplet; correspondingly, the converging distance of the sulfur microlenses can be tuned linearly over a large range (40–90 µm) (Fig. 4e). This selective sulfur growth is amenable to large-scale micro-fabrication and thus offers great potential in creating arrays of tunable optical components.

The effect of different concentration of polysulfides (3 M Li_2_S_8_) and the different solvents (dimethyl sulfoxide (DMSO) and tetraethylene glycol dimethyl ether (TEGDME)) on the wetting behavior and lens performance were also investigated. Similar wetting phenomenon was observed for 3 M Li_2_S_8_ when applying a voltage of 4.0 V (Supplementary Fig. 15). The converging distances of the sulfur microlenses formed by 3 M Li_2_S_8_ were similar to the case of 0.5 M Li_2_S_8_ and could be tuned almost linearly over a range of 45–85 µm (Supplementary Fig. 15b). With DMSO as the solvent, the wetting behavior was observed with a relatively large applied voltage of 5.0 V (Supplementary Fig. 16). The sulfur droplets also changed back to a sphere after the voltage was released (Supplementary Fig. 16d). Because a higher voltage of 5.0 V was applied, there was a larger driving force for the electric field driven wetting in DMSO solvent, the corresponding converging distances of the sulfur microlenses (48–120 µm) were larger than those generated from the 0.5 M Li_2_S_8_ with the DOL/DME solvent (Supplementary Fig. 17). TEGDME solvent has a high viscosity compared with the DOL/DME solvent, so very few sulfur droplets were generated and no obvious wetting behavior was observed with an applied voltage of 4.0 V (Supplementary Fig. 18); therefore, no functional microlenses were created in TEGDME. Based on the above analysis, the concentration of the Li_2_S_8_ electrolyte has minor influence on the performance of the microlenses while the solvents alter significantly the generation and wetting behaviors of the sulfur droplets and therefore have large influence on the lens properties. We believe the condition of 0.5 M Li_2_S_8_ electrolyte with DOL/DME as the solvent is the optimized one for creating the microlenses.

Imaging of the sulfur droplet microlenses is also tested. Arrays of “S” shapes are patterned on the cover glass to serve as imaging objects, which are projected as images above the droplets (Fig. 4f–i). The Gaussian form of the lens equation states:

1 $$\frac{1}{f} = \frac{1}{u} + \frac{1}{v} = \frac{1}{{z_1 - \Delta h}} + \frac{1}{{z_2 - \Delta h}}$$

where *f* is the focal length, *Z*_1_ is the distance from the object to the substrate and *Z*_2_ is the distance from the image to the substrate, with $z_1 - \Delta h$ and $z_2 - \Delta h$ being the object distance and image distance respectively. The distance of the principal plane above substrate $\Delta h$ is calculated to be 4% of the converging distance, which is 1.3 µm in the case of a 60-µm-diameter gold disk. To demonstrate the functionalities of either magnifying or reducing images, we vary the diameter of the microdroplet to change the focal length *f*. In our experiment, the object is set at 121 µm above the substrate (distance between the bottom surface of the cover glass to the liquid sulfur droplet). By tuning the focal length *f* from 52 µm to 59 µm to 67 µm, we change the value of *Z*_2_ from 94 µm to 122 µm to 162 µm, corresponding to a reduced (0.77×), equal (1.00×), and magnified (1.34×) image, respectively (see the schematic drawing in Fig. 4g–i). Based on our model of the droplet (Supplementary Note 2), the values of *Z*_2_ are 92 µm to 119 µm to 156 µm in the above cases, which matches well with the experimental data. While our droplet lens may not strictly satisfy the paraxial approximation, changes in the focal length calculated by our simple model in Supplementary Note 1 are shown to adequately predict the differential focal properties of these lenses, which also agrees well with results obtained by ray tracing.

The sulfur microlens demonstrated here has an operating voltage as low as 3.0–4.0 V, an order of magnitude lower than other electrowetting driven or micro-electromechanical systems (MEMS)-based microlenses, which generally have a high operating voltage of 10–100 V^31,32^. In addition, we demonstrated a wide tunability of the focal length ranging from 30 to 90 µm. In fact, the focal length of our microlens can be readily tuned to longer focal length as we increase the Au disk size. In contrast, the focal length of other microlenses are generally above 500 µm, or above mm and tunable to infinite^2,31,32^. In addition, the sulfur microlens features a big numerical aperture 0.74–0.76, much higher than that of the microlenses reported recently^2^. Finally, the sulfur microlens can be patterned in flexible sizes with tunable focal lengths, amenable to integration into microfluidic devices and other on-chip devices. Overall, this work demonstrates a very promising direction in microlenses, with low operating voltage, large range of focal length, and high numerical aperture.

## Discussion

In summary, we have developed a platform to directly visualize liquid dynamics and wetting behaviors in real time. We have investigated the nucleation and growth behaviors of sulfur droplets and revealed the linear relationship between radius and inverse overpotential, and between the number density of nuclei and the cubic power of the overpotential, in good agreement with classical nucleation and growth theory^21,22^. We demonstrate electrowetting and rapid de-wetting of sulfur droplets under different overpotentials and show different overpotential regimes with signatures of no sulfur, sulfur nucleation, and sulfur wetting. We further realize selective wetting of sulfur droplets on different substrates and explain these phenomena by DFT theoretical calculations. Finally, we demonstrate in situ modulation of liquid sulfur microdroplets and show dynamically tunable microlenses based on liquid sulfur droplets. This platform provides a path to tune the properties of such supercooled liquid materials in real time as well as opens up new approaches for investigating the mechanisms in complex electrochemical reactions.

## Methods

### Fabrication of electrode

Gold electrodes were patterned by depositing Ti/Au (3/50 nm) onto SiO_2_/Si substrates through a deposition mask. For the microlens devices, the gold pads and the “S” patterns on the cover glass were defined by photolithography followed by Ti/Au (3/50 nm) depositions.

### Cell fabrication

The entire cell was assembled inside an Ar-filled glovebox. Patterned gold electrode on the SiO_2_/Si substrates was used as the working electrode. Li metal was laminated onto the copper foil and used as the counter electrode. A cover glass slide was placed on top of the electrodes and the cell was then sealed by a thermoplastic ionomer (Meltonix 1170-60, Solaronix), leaving two small openings for filling liquid electrolyte. Here, Li_2_S_8_ dissolved in DOL/DME (1:1 v/v) with 1 M LiTFSI and 1 wt% LiNO_3_ additive was used as the catholyte. 0.2 M Li_2_S_8_ was used for the sulfur nucleation experiment in Fig. 1 to clearly show the evolution process and 0.5 M Li_2_S_8_ was used for all other experiments. After filling the electrolyte, epoxy was used to finally seal the remaining two openings.

### In situ optical observation of liquid sulfur droplets

The in situ electrochemical reaction was performed with a MTI 8-channel battery tester, while being imaged at the same time using light microscope equipped with air-immersion objective (LMPLFLN-BD, Olympus, ×50, NA 0.5, WD 10.6 mm), broadband Xenon lamp, and CMOS detector. The image series were taken with a frame rate of 1 frame/second and the spatial resolution of the microscope is ~500 nm. Constant voltage charging was used to produce sulfur droplets and wetting phenomena, with galvanostatic discharging to de-wet and dissolve the liquid sulfur.

### Optical microlens characterization

Imaging and measurements of the sample were implemented with an optical microscope coupled to a CCD camera (Acton Pixis1024, Princeton Instruments). The intensity profile of lens focusing was measured with a motor stage with a resolution in the *z* direction of 1 µm (± 50 nm).

### Theoretical calculations

All calculations were performed using DFT as implemented in the Vienna Ab-initio Simulation Package (VASP) code^33,34^. The exchange-correlation interactions were treated by the generalized gradient approximation^35^ in the form of the Perdew–Burke–Ernzerhof functional^36^. The van der Waals interactions were described by using the empirical correction in Grimme’s scheme, i.e., DFT + D_3_^37^. To get the exact band gap of TiO_2_, the Hubbard U (DFT + U) treatment was applied and the U was set to 5.3 eV.

## Supplementary information

Supplementary Information

Description of Additional Supplementary Files

Supplementary Movie 1

Supplementary Movie 2

Supplementary Movie 3

Supplementary Movie 4

Supplementary Movie 5

## Data Availability

The data that support the plots within this paper and other findings of this study are available from the corresponding author upon reasonable request.

## References

[1] Montelongo Y (2017). Electrotunable nanoplasmonic liquid mirror. Nat. Mater..

[2] Nagelberg S (2017). Reconfigurable and responsive droplet-based compound micro-lenses. Nat. Commun..

[3] Teh S-Y, Lin R, Hung L-H, Lee AP (2008). Droplet microfluidics. Lab Chip.

[4] Martines E (2005). Superhydrophobicity and superhydrophilicity of regular nanopatterns. Nano Lett..

[5] Chu K-H, Xiao R, Wang EN (2010). Uni-directional liquid spreading on asymmetric nanostructured surfaces. Nat. Mater..

[6] Park K-C (2012). Nanotextured silica surfaces with robust superhydrophobicity and omnidirectional broadband supertransmissivity. ACS Nano.

[7] Zarzar LD, Kim P, Aizenberg J (2011). Bio-inspired design of submerged hydrogel-actuated polymer microstructures operating in response to pH. Adv. Mater..

[8] He X (2012). Synthetic homeostatic materials with chemo-mechano-chemical self-regulation. Nature.

[9] Zhu Y, Antao DS, Xiao R, Wang EN (2014). Real-time manipulation with magnetically tunable structures. Adv. Mater..

[10] Hayes RA, Feenstra BJ (2003). Video-speed electronic paper based on electrowetting. Nature.

[11] Frieder M, Jean-Christophe B (2005). Electrowetting: from basics to applications. J. Phys.: Condens. Matter.

[12] Cousens NEA, Kucernak ARJ (2017). Reversible ultralow-voltage liquid-liquid electrowetting without a dielectric layer. Faraday Discuss..

[13] Seyrat E, Hayes RA (2001). Amorphous fluoropolymers as insulators for reversible low-voltage electrowetting. J. Appl. Phys..

[14] Moon H, Cho SK, Garrell RL, Kim C-JC (2002). Low voltage electrowetting-on-dielectric. J. Appl. Phys..

[15] Kornyshev AA (2010). Ultra-low-voltage electrowetting. J. Phys. Chem. C..

[16] Lomax DJ (2016). Ultra-low voltage electrowetting using graphite surfaces. Soft Matter.

[17] Zhang G, Walker M, Unwin PR (2016). Low-voltage voltammetric electrowetting of graphite surfaces by ion intercalation/deintercalation. Langmuir.

[18] Liu Nian, Zhou Guangmin, Yang Ankun, Yu Xiaoyun, Shi Feifei, Sun Jie, Zhang Jinsong, Liu Bofei, Wu Chun-Lan, Tao Xinyong, Sun Yongming, Cui Yi, Chu Steven (2019). Direct electrochemical generation of supercooled sulfur microdroplets well below their melting temperature. Proceedings of the National Academy of Sciences.

[19] Liu Y, Zhou G, Liu K, Cui Y (2017). Design of complex nanomaterials for energy storage: past success and future opportunity. Acc. Chem. Res..

[20] Jin Y (2017). Reactivation of dead sulfide species in lithium polysulfide flow battery for grid scale energy storage. Nat. Commun..

[21] Kashchiev D (1982). On the relation between nucleation work, nucleus size, and nucleation rate. J. Chem. Phys..

[22] Oxtoby DW, Kashchiev D (1994). A general relation between the nucleation work and the size of the nucleus in multicomponent nucleation. J. Chem. Phys..

[23] Aurbach D (2009). On the surface chemical aspects of very high energy density, rechargeable Li-sulfur batteries. J. Electrochem. Soc..

[24] Pei A, Zheng G, Shi F, Li Y, Cui Y (2017). Nanoscale nucleation and growth of electrodeposited lithium metal. Nano Lett..

[25] Ely DR, García RE (2013). Heterogeneous nucleation and growth of lithium electrodeposits on negative electrodes. J. Electrochem. Soc..

[26] Sano H, Sakaebe H, Senoh H, Matsumoto H (2014). Effect of current density on morphology of lithium electrodeposited in ionic liquid-based electrolytes. J. Electrochem. Soc..

[27] Feng XJ, Jiang L (2006). Design and creation of superwetting/antiwetting surfaces. Adv. Mater..

[28] Emsley, J. *“Titanium”. Nature’s Building Blocks: An A-Z Guide to the Elements*. 734 (Oxford University Press, Oxford, 2001).

[29] Murade C, Van Der Ende D, Mugele F (2012). High speed adaptive liquid microlens array. Opt. Express.

[30] Kuiper S, Hendriks BHW (2004). Variable-focus liquid lens for miniature cameras. Appl. Phys. Lett..

[31] Krogmann F, Mönch W, Zappe H (2006). A MEMS-based variable micro-lens system. J. Opt. A: Pure Appl. Opt..

[32] Li C, Jiang H (2012). Electrowetting-driven variable-focus microlens on flexible surfaces. Appl. Phys. Lett..

[33] Kresse G, Furthmüller J (1996). Efficient iterative schemes for ab initio total-energy calculations using a plane-wave basis set. Phys. Rev. B.

[34] Kresse G, Furthmüller J (1996). Efficiency of ab-initio total energy calculations for metals and semiconductors using a plane-wave basis set. Comput. Mater. Sci..

[35] Perdew JP, Burke K, Ernzerhof M (1996). Generalized gradient approximation made simple. Phys. Rev. Lett..

[36] Perdew JP, Ernzerhof M, Burke K (1996). Rationale for mixing exact exchange with density functional approximations. J. Chem. Phys..

[37] Grimme S (2006). Semiempirical GGA-type density functional constructed with a long-range dispersion correction. J. Comput. Chem..

