# Supplementary material for: Electrotunable liquid sulfur microdroplets
Source: Nat Commun. 2020 Jan 30;11:606. doi: 10.1038/s41467-020-14438-2 (PMC6992759; doi:10.1038/s41467-020-14438-2)
Supplement: Supplementary file 1 — Supplementary Information [file 41467_2020_14438_MOESM1_ESM.pdf]

Supplementary Information

Electrotunable liquid sulfur microdroplets

Zhou et al.

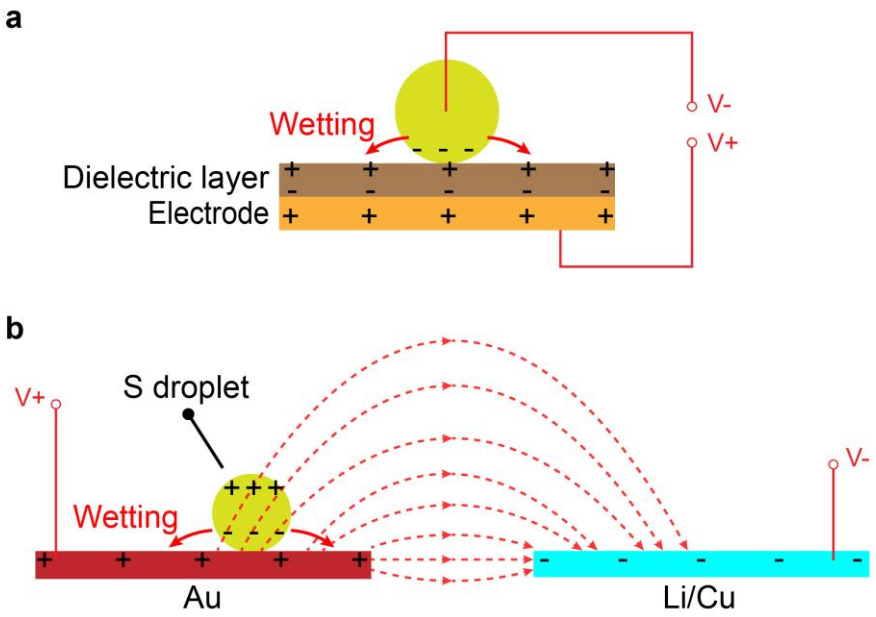

Supplementary Figure 1. (a) Conventional electrowetting set-up with a dielectric layer. (b) A new mechanism to tune wetting of a liquid sulfur droplet directly on a conducting substrate without a dielectric layer.

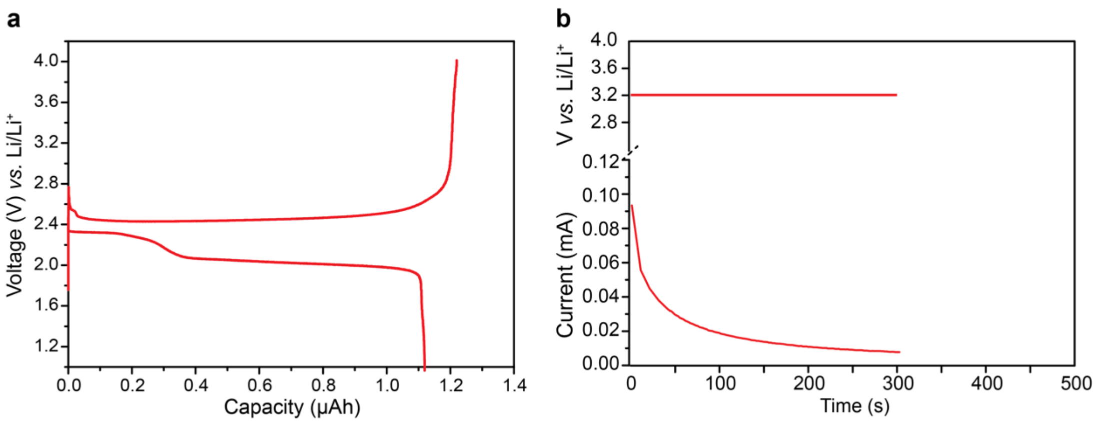

Supplementary Figure 2. (a) Charge/discharge voltage profiles of the electrode at a current of 5 μA showing an equilibrium potential of about 2.4 V. (b) Plot of applying a constant voltage of 3.2 V (η = 0.8 V) with the corresponding current versus time curve for the nucleation and growth of the sulfur droplet on gold.

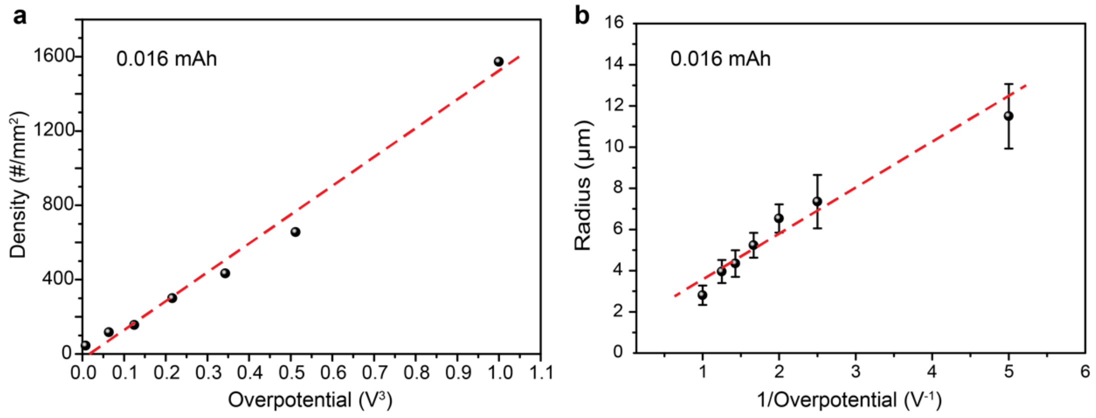

Supplementary Figure 3. (a) Plot of sulfur droplet density versus cubic overpotential (η^3) of sulfur formation at a capacity of 0.016 mAh. (b) Plot of sulfur droplet radius versus inverse overpotential ($1/\eta$) of sulfur formation at a capacity of 0.016 mAh. The red lines show linear fits.

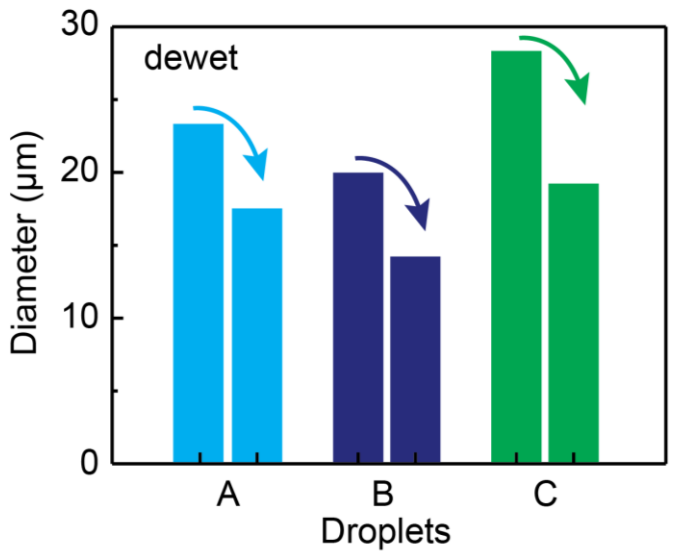

Supplementary Figure 4. Diameter dependence of different sulfur droplets showing the decrease in size after dewetting.

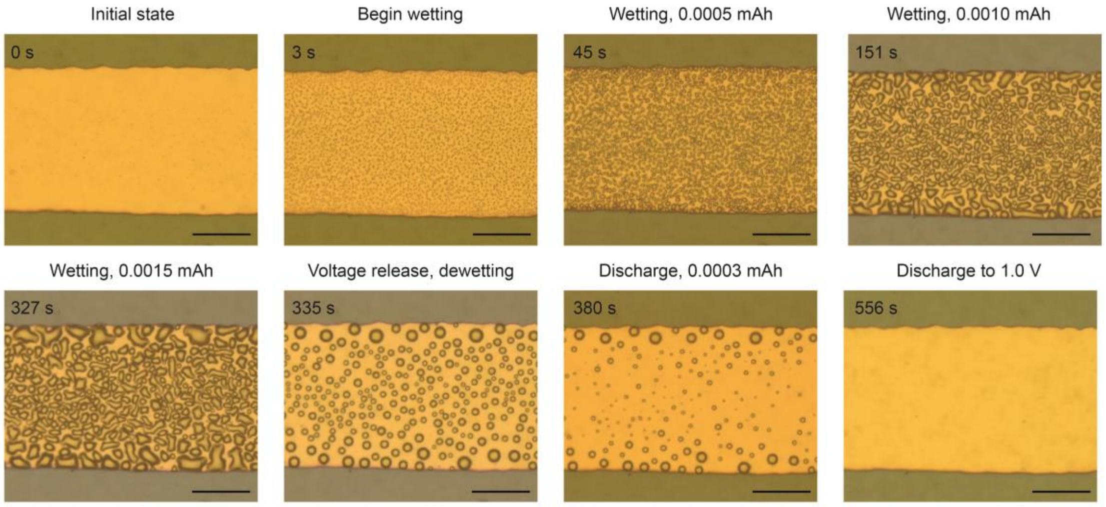

Supplementary Figure 5. Snapshots of sulfur droplet wetting on gold substrate at 4.0 V for a charge capacity of 0.0015 mAh and de-wetting process for discharging to 1.0 V at a current of 0.05 mA. Scale bar is 50 μm.

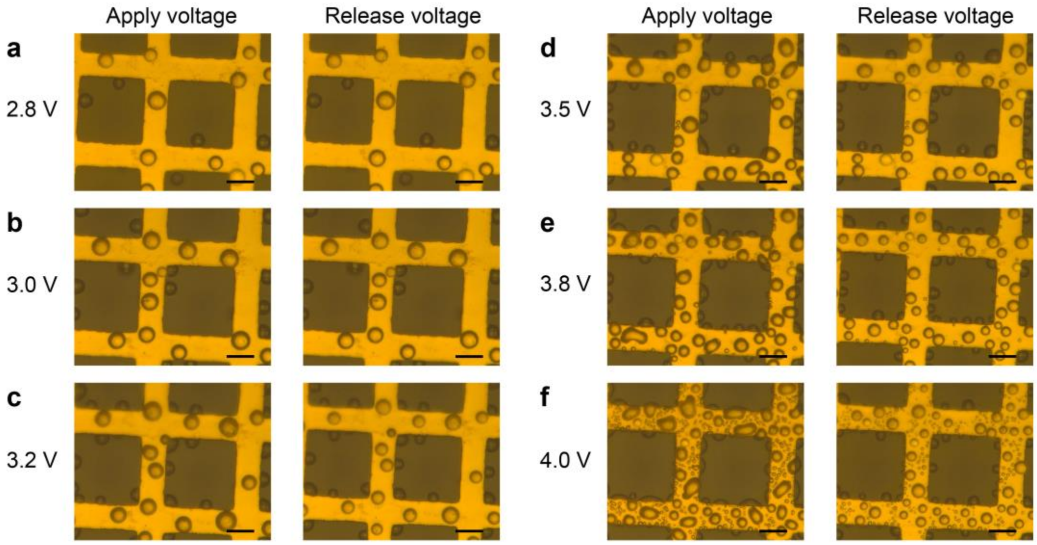

Supplementary Figure 6. The optical images of sulfur droplets generated on a gold-coated nickel TEM grid to obtain the side views of the liquid sulfur droplets. The electrode was held and then released the voltage at (a) 2.8 V, (b) 3.0 V, (c) 3.2 V, (d) 3.5 V, (e) 3.8 V, and (f) 4.0 V in 0.5 M Li_2S_8 electrolyte with DOL/DME as the solvent. Scale bar is 20 μm.

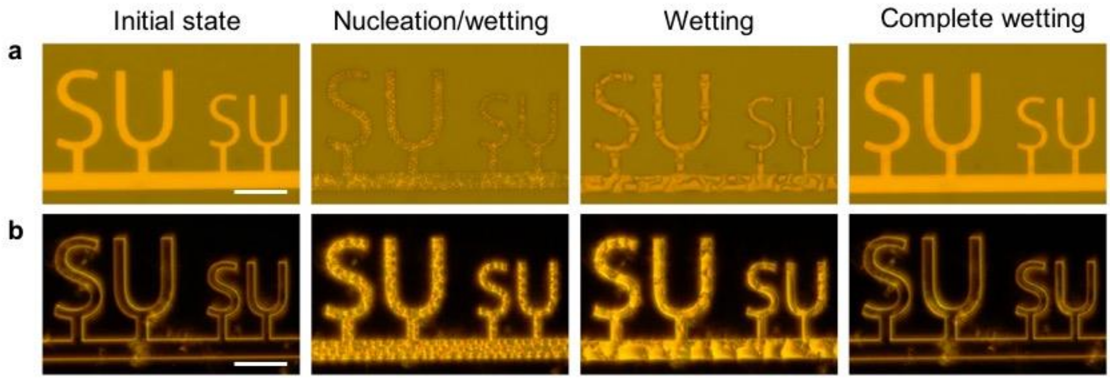

Supplementary Figure 7. (a) Bright field and (b) dark field of designed "SU" pattern illustrating the electro-wetting processes of liquid sulfur on gold. Scale bar is 20 μm.

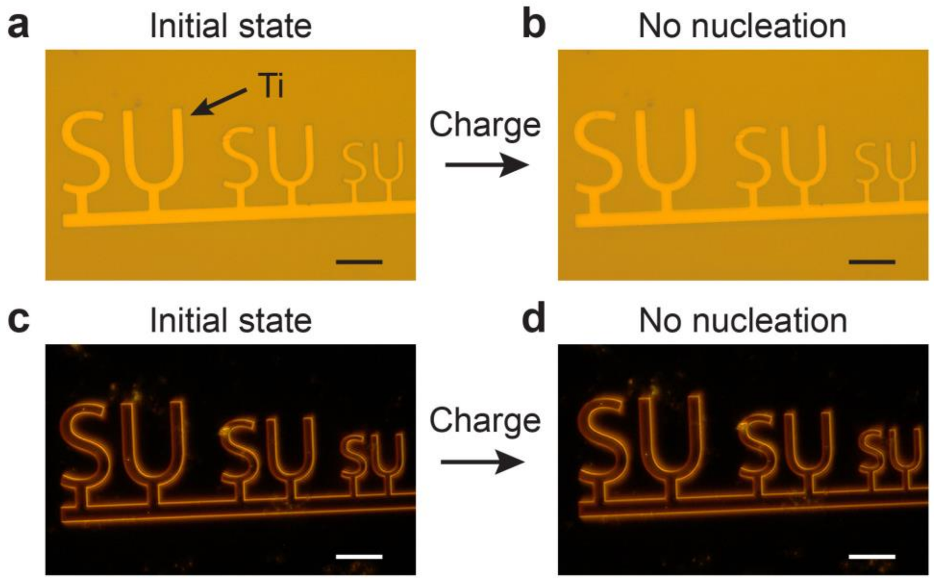

Supplementary Figure 8. (a, b) Bright field and (c, d) dark field of designed "SU" pattern illustrating that sulfur does not wet on titanium surfaces. Scale bar is 20 μm.

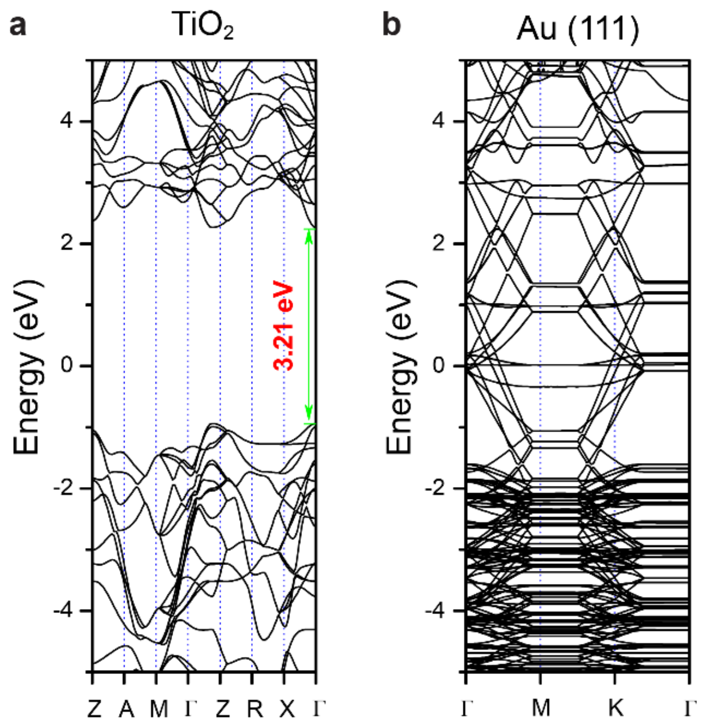

Supplementary Figure 9. Band structures of (a) anatase TiO_2 and (b) gold (111).

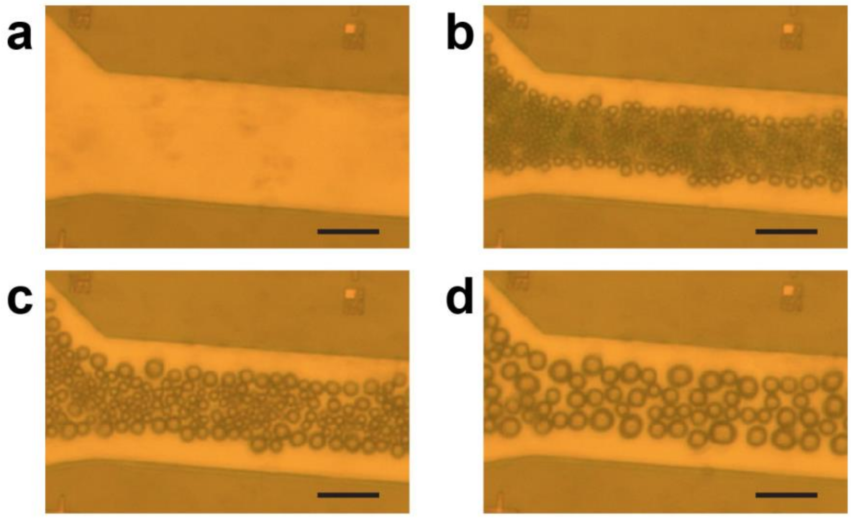

Supplementary Figure 10. (a-d) Optical images of sulfur droplets produced on the surface of titanium at 4.5 V. Scale bar is 10 μm.

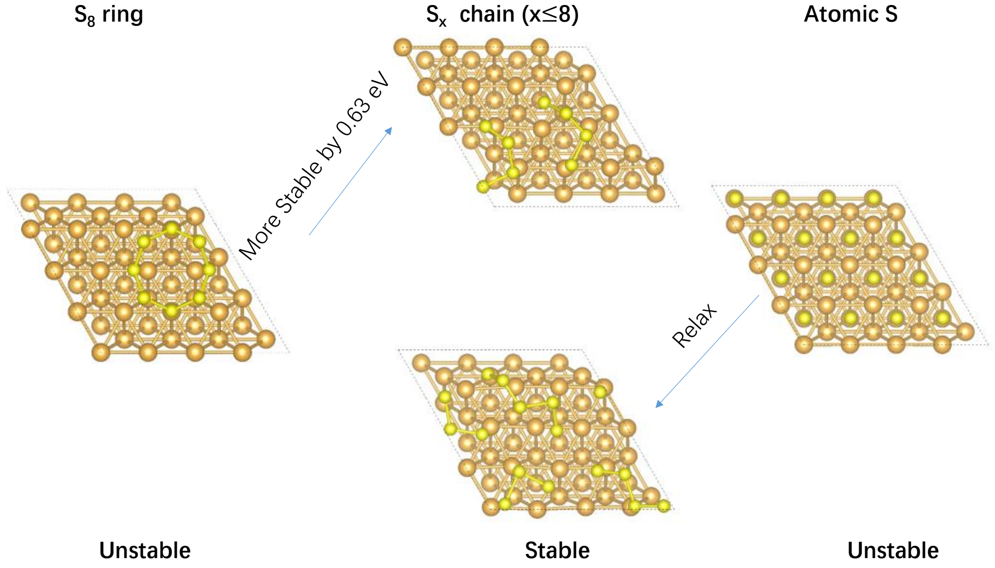

Supplementary Figure 11. DFT calculations of stable sulfur configurations adsorbed on the surface of gold (111). Color codes for the elements: Yellow, S; Gold, Au.

Supplementary Note 1: Measuring the refractive index of liquid electrolyte

A square container with a cavity length of a is placed in the beam path, the cavity is rotated θ with respect to the incident ray (**Supplementary Figure 12**). A lateral displacement of Δy is measured with respect to the light path without the container. Compare the displacement with cavity filled with air Δy_{air} and with the liquid electrolyte Δy_e. The index of the liquid electrolyte n_e meets the following Supplementary Equation 1

$$\begin{cases} \sin\theta = n_e \cdot \sin\theta_e \\ \sin(\theta - \theta_e) \cdot a/\cos\theta_e = \Delta y_e - \Delta y_{air} \end{cases} \quad (1)$$

from which n_e is measured to be 1.4.

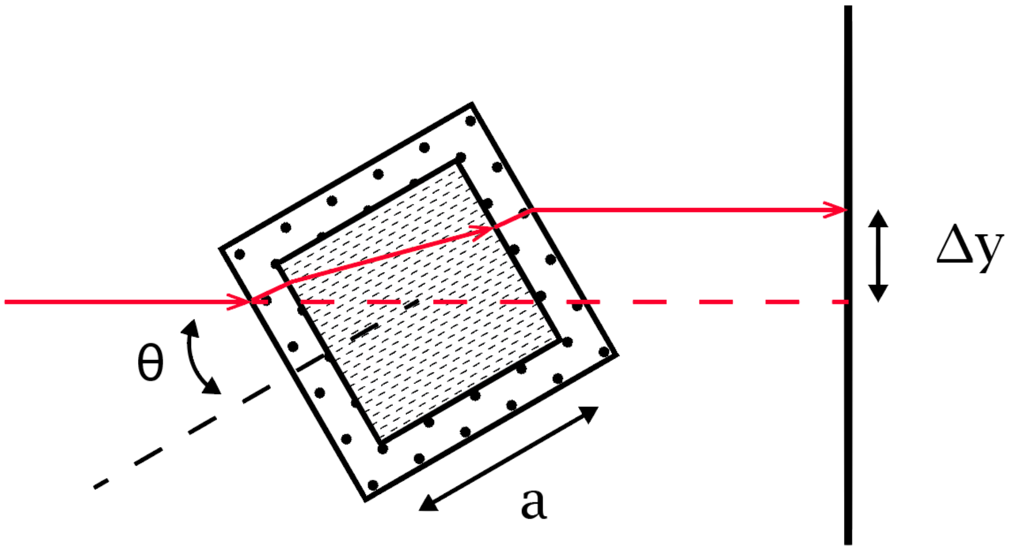

Supplementary Figure 12. Schematic of measuring the refractive index of liquid electrolyte.

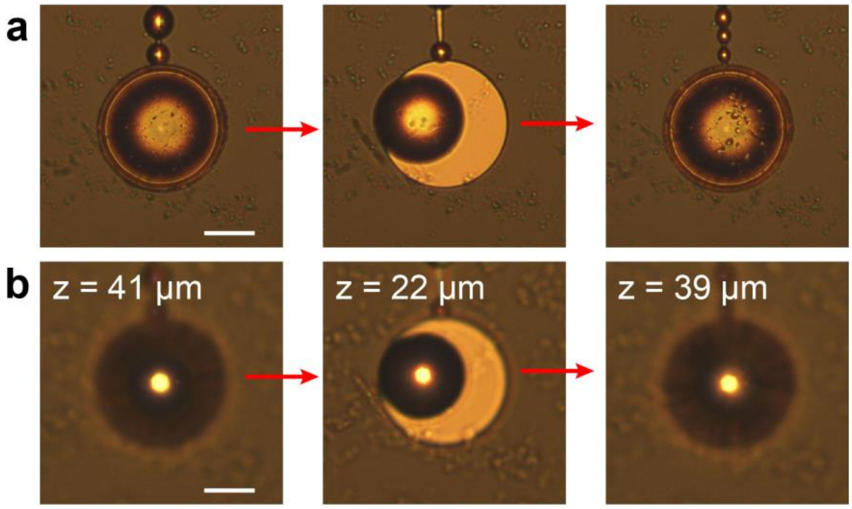

Supplementary Figure 13. (a) The shape of the liquid sulfur droplets can be reconfigured to switch between two different states and (b) the converging distance of the microlens can be correspondingly tuned. Scale bar is 20 μm.

Supplementary Note 2: Measuring the shape and refractive index of sulfur droplets

With the size of the droplet below 100 μm, gravitational force can be negligible compared to surface tension. We assume here the shape of the droplet will always be a spherical cap, as in **Supplementary Figure 14**. A sphere is cut by the plane of substrate, the part above the substrate is a sphere, with $\cos\theta$ ranging from 1 to -1. From observation (**Supplementary Figure 13**), when a voltage is applied to the gold disk, the cross section of the sulfur droplets has the same size as the patterned gold disk. When the voltage is released, the cross section will be reduced. But for both cases, the volume of the droplet does not change. So

$$\frac{\pi}{3}{R_{wet}}^3(2+\cos\theta_{wet})(1-\cos\theta_{wet})^2 = \frac{\pi}{3}{R_{unwet}}^3(2+\cos\theta_{unwet})(1-\cos\theta_{unwet})^2 \quad (2)$$

Since the droplet bottom overlaps with the gold disk, when voltage applied, we have

$$2R_{wet}\sin\theta_{wet} = D_{disk} \quad (3)$$

Now we resort to focal points to help us determine the index of sulfur, set $\alpha = n_s/n_e$. Under the paraxial assumption,

$$z_{converge} = R(1-\sin\theta) + \frac{(1+1/(\alpha-1)) - 2(1-\sin\theta)}{2[\alpha-(\alpha-1)(1-\sin\theta)]}\cdot R \quad (4)$$

R_{unwet} can be measured from experiment. Since the droplet will have a contact angle greater than 90 degrees when it is not wetting, we start from assuming the droplet is a whole sphere without voltage, which means $\cos\theta_{unwet} = -1$. Supplementary Equation 2 and Supplementary Equation 3 give us R_{wet} and θ_{wet}. Then we match Supplementary Equation 4 with the measured converging distance, which determines

α. Then α is set as a fixed value when using Supplementary Equation 4 for the unwetted droplet, and we deduce a new θ_{unwet}. We iterate the process above until the variables all converge.

Comparing the paraxial calculation with ray tracing, the results are the same, demonstrating that the spherical aberration is a negligible perturbation of the paraxial condition for the calculation above.

For all the sizes of gold disks we measured, the results are the same, with

$$\begin{cases} \alpha = 1.45 \pm 0.05 \\ \cos\theta_{wet} = 0.18 \pm 0.05 \\ \cos\theta_{unwet} = -0.85 \pm 0.05 \end{cases} \quad (5)$$

Since $\alpha = n_s/n_e$ and is measured to be 1.45, and n_e measured to be 1.4, we have $n_s = 2.0$. This matches well with the experimental data for molten sulfur above 100 ℃.[1,2] The small difference is likely due to the temperature difference, since our experiment is carried out in room temperature.

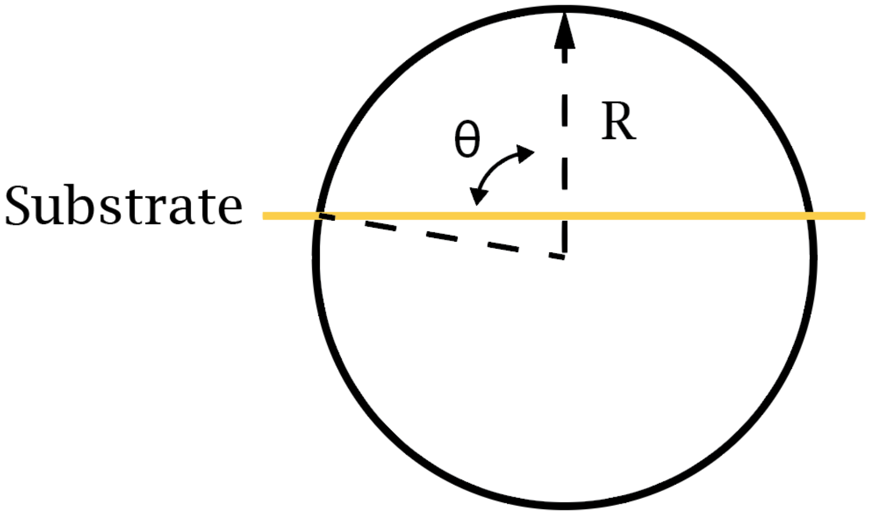

Supplementary Figure 14. Schematic of a spherical cap.

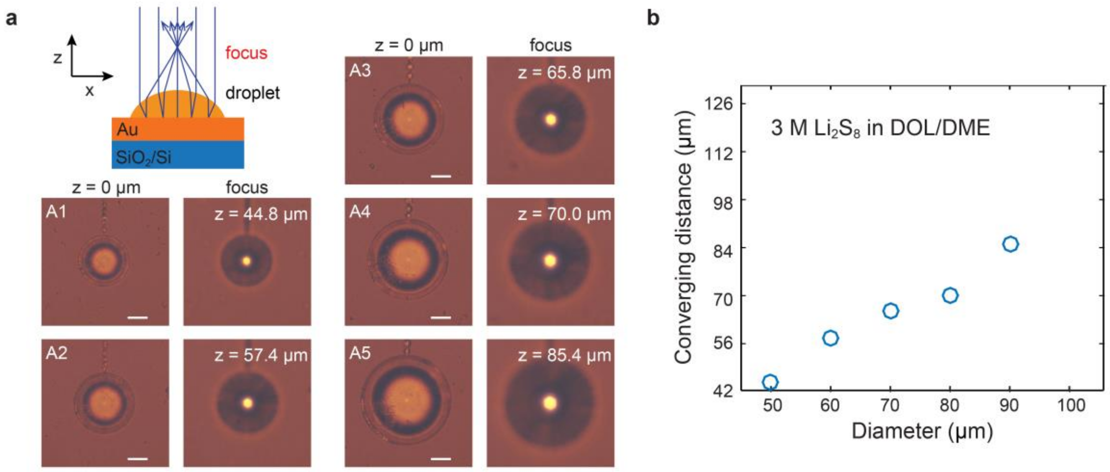

Supplementary Figure 15. (a, b) The converging distance of the microlenses can be tuned as a function of the diameter of the sulfur droplets in 3 M Li_2S_8 electrolyte with DOL/DME as the solvent. Scale bar is 20 μm.

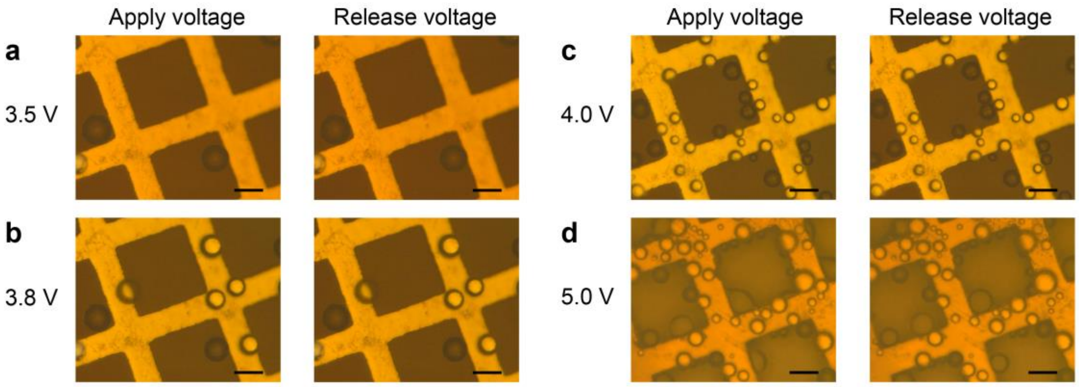

Supplementary Figure 16. The optical images of sulfur droplets generated on a gold-coated nickel TEM grid to obtain the side views of the liquid sulfur droplets. The electrode voltage was held and then released at (a) 3.5 V, (b) 3.8 V, (c) 4.0 V, and (d) 5.0 V in 0.5 M Li_2S_8 electrolyte with DMSO as the solvent. Scale bar is 20 μm.

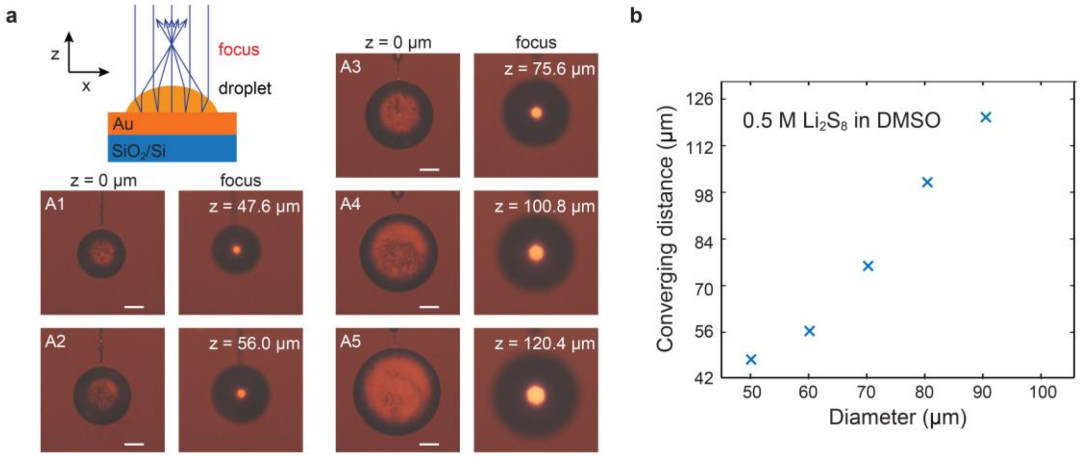

Supplementary Figure 17. (a, b) The converging distance of the microlenses can be tuned as a function of the diameter of the sulfur droplets in 0.5 M Li_2S_8 electrolyte with DMSO as the solvent. Scale bar is 20 μm.

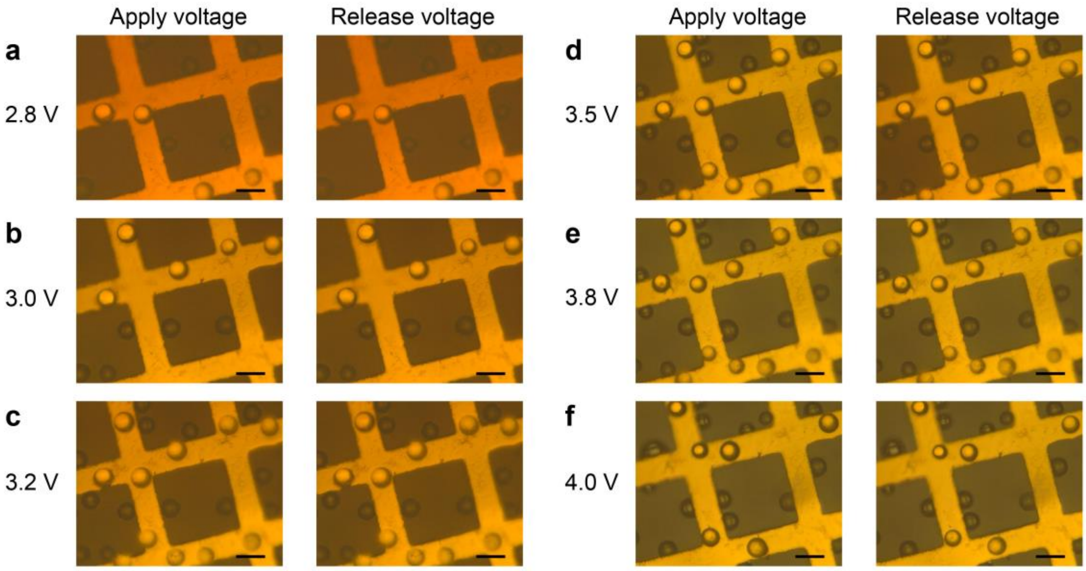

Supplementary Figure 18. The optical images of sulfur droplets generated on a gold-coated nickel TEM grid to obtain the side views of the liquid sulfur droplets. The electrode voltage was held and then released at (a) 2.8 V, (b) 3.0 V, (c) 3.2 V, (d) 3.5 V, (e) 3.8 V, and (f) 4.0 V in 0.5 M Li_2S_8 electrolyte with TEGDME as the solvent. Scale bar is 20 μm.

Supplementary References

1 Sasson, R. & Arakawa, E. Temperature dependence of index of refraction, reflection, and extinction coefficient of liquid sulfur in the 0.4–2.0-μm wavelength range. *Appl. Opt.* **25**, 2675-2680, (1986).

2 Donaldson, A. & Caplin, A. Refractive index of molten sulphur. *Philos. Mag. B* **52**, 185-197, (1985).
