# Supplementary material for: Electrotunable liquid sulfur microdroplets
Source: Nat Commun. 2020 Jan 30;11:606. doi: 10.1038/s41467-020-14438-2 (PMC6992759; doi:10.1038/s41467-020-14438-2)
Supplement: Supplementary file 2 — Description of Additional Supplementary Files [file 41467_2020_14438_MOESM2_ESM.pdf]

Description of Additional Supplementary Files

File Name: Supplementary Movie 1

Description: Sulphur droplet nucleation, growth, and dissolution (discharge) processes at 2.8 V on gold substrate with the overpotential of 0.4 V. Play speed is 15x of the actual speed.

File Name: Supplementary Movie 2

Description: Sulphur droplet nucleation, growth, and dissolution (discharge) processes at 3.4 V on gold substrate with the overpotential of 1.0 V. Play speed is 15x of the actual speed.

File Name: Supplementary Movie 3

Description: Real time imaging for the wetting, merging, growth and de-wetting processes on gold substrate. The electrochemical cell was charged first at constant voltage 3.2 V, and then galvanostatically discharged to 1.5 V. Play speed is 5x of the actual speed.

File Name: Supplementary Movie 4

Description: Real time sulphur droplet wetting, dewetting and dissolution (discharge) on gold substrate. Wetting: charge at 4.0 V for a charge capacity of 0.0015 mAh, de-wetting: discharge to 1.0 V at a current of 0.05 mA. Play speed is 25x of the actual speed.

File Name: Supplementary Movie 5

Description: “SU” pattern with alternate gold and titanium design illustrating substrate-dependent electrowetting of sulphur. Play speed is 2x of the actual speed.
